# Novel Peptide Conjugates of Modified Oligonucleotides for Inhibition of Bacterial RNase P

**DOI:** 10.3389/fphar.2019.00813

**Published:** 2019-07-19

**Authors:** Darya Novopashina, Mariya Vorobyeva, Anton Nazarov, Anna Davydova, Nikolay Danilin, Lyudmila Koroleva, Andrey Matveev, Alevtina Bardasheva, Nina Tikunova, Maxim Kupryushkin, Dmitrii Pyshnyi, Sidney Altman, Alya Venyaminova

**Affiliations:** ^1^Institute of Chemical Biology and Fundamental Medicine, Siberian Branch of the Russian Academy of Sciences, Novosibirsk, Russia; ^2^Department of Natural Sciences, Novosibirsk State University, Novosibirsk, Russia; ^3^Shemyakin-Ovchinnikov Institute of Bioorganic Chemistry, Russian Academy of Sciences, Moscow, Russia; ^4^Department of Molecular, Cellular and Developmental Biology, Yale University, New Haven, CT, United States; ^5^Division of Life Sciences, Arizona State University, Tempe, AZ, United States

**Keywords:** bacterial RNase P, inhibition of RNase P, modified oligonucleotides, oligo(2’-*O*-methylribonucleotides), phosphoryl guanidine oligonucleotides, peptide conjugates of oligonucleotides, penetration into bacterial cells, antibacterial activity

## Abstract

Novel alternatives to traditional antibiotics are now of great demand for the successful treatment of microbial infections. Here, we present the engineering and properties of new oligonucleotide inhibitors of RNase P, an essential bacterial enzyme. The series of 2’-*O*-methyl RNA (2’-OMe-RNA) and phosphoryl guanidine oligonucleotides were targeted to the substrate-binding region of M1 RNA subunit of the RNase P. Uniformly modified 2’-OMe RNA and selectively modified phosphoryl guanidine oligonucleotides possessed good stability in biological media and effectively inhibited RNase P. Their conjugates with transporting peptides were shown to penetrate bacterial cells (*Escherichia coli* and *Acinetobacter baumannii*) and inhibit bacterial growth.

## Introduction

The design of novel compounds with antibacterial activity is one of the most acute issues of modern chemical biology, biotechnology, and medicine. Despite a broad spectrum of antimicrobial agents, the problems of the drug resistance and side effects remain unsolved until now (Guidry et al., 2014; Llor and Bjerrum, 2014). A promising strategy to overcome these problems could be a displacement of low-molecular-weight antimicrobial compounds targeting essential bacterial biomolecules and pathways for nucleic acid-based therapeutics targeting bacterial nucleic acids. Within this context, the development of oligonucleotides that specifically interact with bacterial RNAs, block their functions, and thereby inhibit bacterial growth is of particular interest (Bai et al., 2010). Bacterial RNase P, a tRNA-processing enzyme, is an attractive target for the design of antibacterial oligonucleotides (Guerrier-Takada et al., 1983; Altman, 2011). On the one side, RNase P is a key player of the well-established EGS (external guide sequence) technology (see, e.g., the reviews Davies-Sala et al., 2015; Derksen et al., 2015). Specially designed EGS oligonucleotides mimicking the 3’-fragment of the pre-tRNA substrate can address the enzyme to cleave the target sequence within specific bacterial mRNA. On the other side, as one of the essential bacterial enzymes, RNase P itself represents an attractive target for antibacterial agents. The enzyme contains an RNA subunit (catalytic M1 RNA), which gives a possibility to inhibit RNase P by complementary oligonucleotides and thus suppress the bacterial growth (Gruegelsiepe et al., 2006). One of the main advantages of this approach is the specific targeting of bacterial cells provided by huge differences between eukaryotic and bacterial enzymes, primarily their M1 RNA sequences (Klemm et al., 2016). Oligonucleotide inhibitors targeted to the bacterial RNase P should not cause any off-target effects on eukaryotic cells. The possibility of bacterial growth suppression by RNA, DNA, locked nucleic acid (LNA), and peptide nucleic acid (PNA) oligonucleotide inhibitors targeting certain M1 RNA regions was demonstrated earlier (Gruegelsiepe et al., 2003; Willkomm et al., 2003; Gruegelsiepe et al., 2006).

With all significant achievements in the design of oligonucleotide inhibitors of RNase P, there is plenty room for improvement of the resistance of these oligonucleotides to nuclease digestion and effectivity of their interaction with the enzyme, as well as penetration into bacterial cells.

Here, we present the design of novel modified oligonucleotides as RNase P inhibitors. A set of 2’-OMe-RNA and selectively modified phosphoryl guanidine oligonucleotides were generated and evaluated for their inhibiting properties. Conjugates of most prominent modified oligonucleotides with cell-penetrating peptides were shown to be capable of penetrating bacterial cells and suppress their growth.

## Materials and Methods

Tris(hydroxymethyl)aminomethane (Sigma-Aldrich, USA), acetonitrile (PanReac, Spain), acrylamide, N,N’-methylenebisacrylamide (Acros Organics, Belgium), sodium perchlorate, ammonium persulfate, “Stains-All” dye, magnesium chloride, urea, xylene cyanol FF, bromophenol blue, potassium chloride (Fluka, Switzerland), Na_2_EDTA (AMRESCO, USA), fetal bovine serum (FBS, heat-inactivated, Invitrogen, USA), culture medium DMEM (Life Technologies, USA), γ-[^32^P]-ATP (120 TBq/mol, «Biosan», Russia), and other reagents and solvents supplied by Sigma-Aldrich, PanReac, and Acros Organics. 3-Maleimidopropanoic acid pentafluorophenyl ester (MPPf) was synthesized by analogy with Kida et al. (2007). Peptides bearing cysteine at N-terminus were obtained from Almabion (Russia): *Pept1*—CKWKLFKKIGAVLKVLTTG, *Pept2*—CRGWEVLKYWWNLLQY, *Pept3*—CHHHHHHHHHHHHHHHH, and *Pept4*—CINVLGILGLLGEALSEL.

C5 protein unit of *Escherichia coli* RNase P was prepared as described in Guerrier-Takada et al. (1983) and kindly provided by Prof. Khodyreva S.N. (ICBFM SB RAS, Novosibirsk, Russia); M1 RNA was synthesized by protocol (Guerrier-Takada et al., 1989) and kindly provided by Prof. Moor N.A. (ICBFM SB RAS, Novosibirsk, Russia). The DH5α strain of *E. coli* and the type ATCC (#19606) strain of *Acinetobacter baumannii* from the collection of thermophilic organisms and type cultures of ICBFM SB RAS were used for investigation of cell penetration and suppression of bacterial growth.

The radioactive 5’-[^32^P]-labeling of oligonucleotides was performed using four MBq [γ-^32^P]-ATP and T4 Polynucleotide Kinase (Thermo Scientific, USA) by standard protocol. The isolation of 5’-[^32^P]-labeled oligonucleotides was performed with Micro Bio-Spin P30 columns (Bio-Rad, USA).

The gels were dried using Gel Dryer B35 instrument (Bio-Rad, USA) and radioautographed using Bio-Rad Exposure Cassette-K and photosensitive Kodak Storage Phosphor Screen SO230 (Bio-Rad, USA). The screen was scanned using Pharos FX (Bio-Rad Laboratories Inc., CA, USA) Phosphorimager; the images acquired were processed using Quantity One Analysis Software (Bio-Rad Laboratories Inc., CA, USA).

Fluorescence was measured in microplates Costar 96-Well Half-Area Black (Thermo Fisher Scientific, USA) using CLARIOstar instrument (BMG LABTECH, USA).

Water filtration system simplicity (Millipore, USA), spectrophotometer NanoDrop 1000 (Thermo Fisher Scientific, USA), thermomixers and centrifuges (Eppendorf, Germany), Speed-Vac Concentrator SVC-100H (Savant, USA), the gel-electrophoresis system (Helicon, Russia), and gel-documentation system Molecular Imager FX (Bio-Rad, USA) were also used.

### Synthesis of Modified Oligonucleotides and Model RNA Target

Synthesis of modified oligonucleotides and model RNA target was carried out by the solid-phase phosphoramidite method on the ASM-800 synthesizer (Biosset, Russia) using protocols optimized for this instrument. 2’-*O*-*tert*-Butyldimethylsilyl (2’-*O*-TBDMS) protected RNA phosphoramidites, 2’-*O*-methyl RNA, and DNA phosphoramidites; solid supports with first nucleosides, modified solid supports for the synthesis of 3’-fluorescein; 3’-BHQ (Black Hole Quencher), and 3’-amino linker (aminohexanol) containing oligonucleotides were purchased from ChemGene (USA). Fluorescein phosphoramidite (Glen Research, USA) was used for the introduction of fluorescein residue on 5’-end of oligomers. DMS(O)MT-protected amino linker C6 (Lumiprobe, Russia) was used to prepare oligonucleotides bearing 5’-amino linker. Phosphoryl guanidine oligonucleotides were prepared in LLC «NooGene» (ဂRussia) using protocols published earlier (ဂKupryushkin et al., 2014; Stetsenko et al., 2014).

All oligonucleotides and their derivatives were deblocked by standard protocols for the corresponding type of modification. Isolation of oligoribonucleotides, their modified analogs, and derivatives was performed using preparative electrophoresis in denaturating 15% PAAG. Oligodeoxyribonucleotides and phosphoryl guanidine oligonucleotides were isolated by high-performance liquid chromatography (HPLC) on Agilent 1 200 HPLC system (Agilent Technologies, USA) using Zorbax SB-C18 (4.6 × 150 mm) column in acetonitrile concentration gradient 0–50% in 20 mM triethylammonium acetate (pH 7.0) during 30 min and rate 2 ml/min.

### Investigation of the Cleavage of Modified Oligonucleotides by Serum Nucleases

The treatment of 5’-[^32^P]-labeled oligonucleotides (**r-inh**, **m-inh**, **d-inh**) by 10% FBS in DMEM was carried out at 37°C. The 5-µl aliquots were taken after 15, 30, 60, 120, 240, and 360 min and 1 day, mixed with Stop Mix solution and analyzed by denaturating 15% polyacrylamide gel electrophoresis (PAGE).

### Mass Spectrometry of Oligonucleotides and Their Peptide Conjugates

The mass spectra of the oligonucleotide conjugates were recorded on a Matrix-Assisted Laser Desorption Ionisation-Time-of-Flight (MALDI-TOF) Autoflex Speed mass-spectrometer (Bruker Daltonics, Germany). The mass spectra of phosphoryl guanidine oligonucleotides were obtained using Electrospray Ionisation Mass Spectrometry (ESI-MS) on the Agilent G6410A LC-MS/MS instrument (Agilent Technologies, USA).

### Hydrolysis of RNA Target by RNase P

The hydrolysis of fluorescent RNA target (5’-**flu**-pGUUUUCUUCGGUGGGGUUUCUUCCCCACCACCA-**BHQ**-3’) at a concentration from 50 to 300 nM was carried out in 50 µl of a solution containing 10 mM Tris-HCl (pH 7.5), 10 mM MgCl_2_, 100 mM NH_4_Cl, 5 nM M1 RNA, and 50 nM of C5 protein at 37°С. RNA target was annealed for 2 min at 90°С and cooled up to 37°С. The RNA target solution was placed in the wells of Costar 96 Half-Area Microplate. The reaction was initiated by addition of the mixture of enzyme and inhibitor oligonucleotide. Fluorescence intensity was registered each minute using CLARIOstar instrument. The excitation wavelength was 483 nm, and the emission wavelength was 530 nm. The data were processed using Mars Data Analysis Software (BMG Labtech, USA).

### Calculation of Kinetic Parameters of Hydrolysis of Fluorescent RNA Target by RNase P in the Presence of Inhibiting Oligonucleotides

The dependence of fluorescence intensity data from time was analyzed using the equation (1) in GraphPad Prism 5.0.4.533 software:

(1) $$F=F_{pl}\left(1-e^{k_{\mathrm{obs}}\cdot t}\right)$$

where *F* is the intensity of fluorescence on 530 nm at the moment *t*, *F* *_p_* is the fluorescence intensity upon stage (stable) phase, *k* *_obs_* is the pseudo-first order rate constant, and *t* is the reaction time. The values of *k* *_obs_* were used to calculate parameters *K*_m_ and *V*_max_ using equation (2) in the same software package.

(2) $$k_{obs}=\frac{V_{\max}}{K_{m}+S}$$

where *V*_max_ is the maximum reaction rate, *K*_m_ is the Michaelis constant, and *S* is the substrate concentration. The *k*_cat_ value was calculated from the relationship *V*_max_ = *k*_cat_ · *E*_0_, where *E*_0_ is the enzyme concentration.

The *IC* *_50_* values were calculated using observed rate constants *k* *_obs_* and equation (3) for the concurrent inhibition in the same software package

(3) $$k_{\mathrm{obs}}=\frac{\left(\frac{\mathrm{Ki}}{K_{m}}\right)V_{\max}}{{\mathrm{IC}}_{50}+I}$$

*k* *_obs_* is the pseudo-first order rate constant, *Vmax* is the maximal rate of reaction, *IC*_50_ is the half-maximal inhibitory concentration, *K* *_i_* is the constant of the inhibition, *K_m_* is the Michaelis constant, and *I* is the concentration of inhibiting oligonucleotide.

### Synthesis of Peptide Conjugates of Inhibiting Oligonucleotides

The solution of MPPf (1 mg, 3 μmol) in 20 μl of DMSO was added to the solution of 5’- or 3’-amino-modified oligonucleotides (**m-inh** or **pgd-inh3**) (120 nmol) in 5 μl of 0.02 M (4-(2-hydroxyethyl)-1-piperazineathanesulfonic acid)(HEPES) (pH 7.2) by portions of 10, 5, and 5 μl each 30 min. The reaction mixture was incubated at 37°C upon mixing at 1,200 rpm. After 30 min from the last addition of MPPf, the reaction mixture was precipitated by 2% NaClO_4_ in acetone, and the pellet was washed by acetone and dried in air. The precipitate was dissolved in 10 μl of 0.01 M HEPES (pH 7.2). The solution of the peptide (***Pept1***, ***Pept2***, ***Pept3***, or ***Pept4***) in 20 μl of dimethylsulfoxide (DMSO) was added to the maleimide-modified oligonucleotide solution, and the reaction was carried out for 1–3 h at 37°C upon mixing at 1,200 rpm. The conjugates were isolated by electrophoresis in denaturating 12% PAAG (acrylamide:bisacrylamide, 30:0,5), eluted by 0.3 М NaClO_4_, and desalted using Amicon 3K (Millipore, USA). Conjugates were precipitated as Na^+^ salts.

### Investigation of Conjugate Penetration to the Bacterial Cells

Cell penetration studies were carried out at the cultures of *E. coli* and *A. baumannii*. The night culture of bacterial cells was diluted at 100 times by growth medium LB (lysogeny broth, Luria-Bertani medium); then, the cells (3–5×10^6^ cells per ml) were incubated for 2 h at 37°C upon swinging up to the optical density OD_600_ = 0.35. The cell culture was prepared at the exponential phase of growth (5–6×10^6^ cells/ml). Then cells were precipitated by centrifugation at 4,000×g for 4 min, and resuspended at LB medium containing peptide conjugate. The final concentrations of conjugates in the medium were 1 or 0.2 µМ. The cells were incubated for 1 h at 37°C upon swinging in the dark. The cells were precipitated by centrifugation at 4,000×g for 4 min; then, 100 μl of 0.9% NaCl solution was added to precipitate, and the procedure was repeated twice. The cell precipitate was resuspended in 100 μl of 4% formaldehyde solution in phosphate buffer and incubated for 30 min at room temperature upon swinging. Then, the cells were washed three times by sterile phosphate buffer and incubated with 4′,6-diamidino-2-phenylindole (DAPI) for additional 15 min.

The slides were prepared by placing 10 μl of cell suspension and 10 μl of antifade diamond solution (Life Technologies, USA) and covering with the 25×25-mm cover glass. Visualization was performed using the confocal laser-scanning microscope LSM 710 Carl Zeiss upon magnification at 630 times and excitation at 405 nm for DAPI and 488 nm for fluorescein isothiocyanate (FITC) (fluorescein). The pictures were analyzed with ZEN 2011 Black Edition software.

Flow cytometry was performed using the NovoCyte Instrument (ACEA Biosciences, USA).

### Bacterial Growth Inhibition Experiments

Oligonucleotides and their conjugates were tested for inhibition of bacterial cell growth using *E. coli* (strain DH5α). Cell cultures were incubated with the oligonucleotide or oligonucleotide-peptide conjugate for 22 h in 96-well plate at 37°C and rotation at 530 rpm. In control samples, water was used instead of the conjugate solution. The growth of cultures was estimated by optical density at 595 nm using plate reader (Uniplan, Russia).

## Results and Discussion

**Figure 1:**
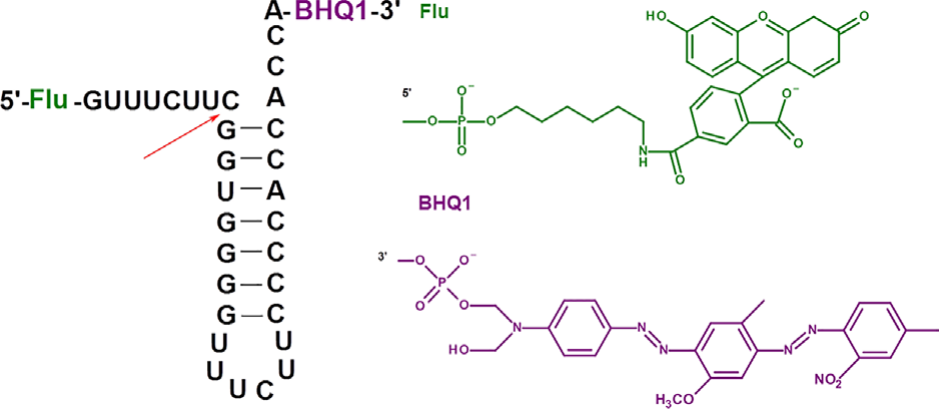
Model fluorescent RNA target for the investigation of inhibition of RNase P. The arrow shows the site of RNA hydrolysis.

To study the RNase P activity in the presence of oligonucleotide inhibitors, we employed the model synthetic hairpin RNA imitating the natural pre-tRNA substrate of RNase P. This RNA contained 5’-fluorescein and 3’-BHQ quencher residues (**Figure 1**).

Upon the RNA hydrolysis, fluorophore and quencher are moving away from each other, and the fluorescence arises. The values of the kinetic parameters for the hydrolysis of fluorescent RNA target by *E. coli* RNase P (*K*_m_ = 83 ± 49 nM, *k*_cat_ = 24 ± 5 min^–1^, see **Figure S1**, **Table S1**) are close to those for the hydrolysis of the native pre-tRNA^Tyr^ (*K*_m_ = 33 nM, *k*_cat_ = 29 min^–1^) (Jiang et al., 2014). Therefore, we validated the appropriate catalytic activity of the enzyme and the feasibility of fluorescent RNA as a model substrate. The presence of fluorescent and quencher groups had no impact on the affinity of the substrate to the enzyme and the cleavage rate.

One of the most suitable sites for RNase P inhibition is the region of P15 loop taking part in recognition of CCA sequence on the 3’-end of the pre-tRNA substrate (Childs et al., 2003; Gruegelsiepe et al., 2003; Willkomm et al., 2003). Modified oligomers complementary to the 291–304 fragment of *E. coli* M1 RNA and containing 2’-*O*-methylated or LNA monomers specifically inhibited RNase P with practically the same IC_50_ values (Childs et al., 2003). PNA conjugates with peptides suppressed *E. coli* growth in cell culture experiments (Gruegelsiepe et al., 2006).

**Figure 2:**
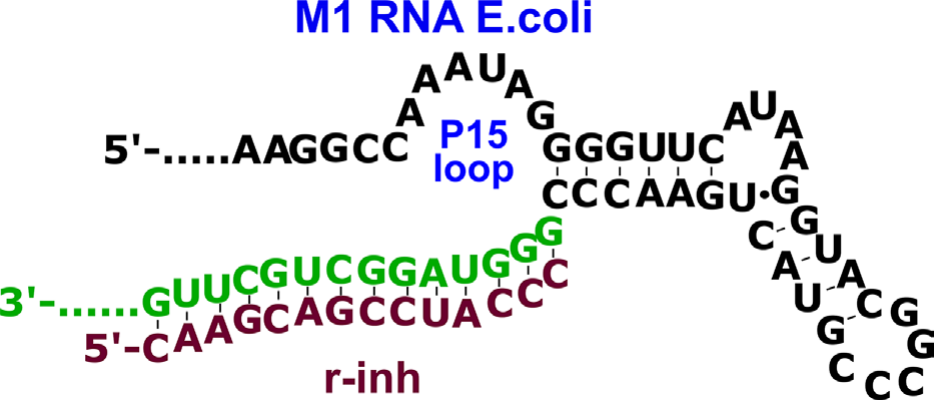
The complex of oligoribonucleotide inhibitor (**r-inh**) with P15 loop region of the M1 RNA *E. coli*.

Based on these data, we designed 14-nt oligonucleotide inhibitors complementary to nucleotides 291–304 in the P15-loop (**Figure 2**, **Table 1**). The set of inhibitors included native oligoribonucleotide, oligo(2’-*O*-methylribonucleotide), oligodeoxyribonucleotide, and oligodeoxyribonucleotides with phosphoryl guanidine modifications in different positions.

**Table 1:** The inhibiting oligonucleotides and IC_50_ values for hydrolysis of RNA target.

Inhibitor	Sequence, 5’-3’	IC_50_, nM
**r-inh**	5’-r(CAAGCAGCCUACCC)	12 ± 4
**m-inh**	5’-C^m^A^m^A^m^G^m^C^m^A^m^G^m^C^m^C^m^U^m^A^m^C^m^C^m^C^m^	90 ± 30
**d-inh**	5’-d(CAAGCAGCCTACCC)	270 ± 90
**pgd-inh1**	5’-d(CxAxAxGxCxAxGxCxCxTxAxCxCxCx)-RNH_2_	No effect
**pgd-inh2**	5’-d(CxAxAxGxCxAxGxCCTACCC)	400 ± 100
**pgd-inh3**	5’-d(CAAGCAGCxCxTxAxCxCxC)	100 ± 40

The cleavage conditions: 5 nM M1 RNA, 50 nM C5 protein, 100 nM fluorescent RNA target, 0-125 nM inhibiting oligonucleotide, 10 mM Tris-HCl, pH 7.5, 10 mM MgCl_2_, 100 mM NH_4_Cl, 37°C. N^m^ – 2’-O-methylribonucleotide; Nx – phosphoryl guanidine deoxyribonucleotide; x - phosphate groups modified with 1,3-dimethylimidazolidine-2-imine residues, RNH_2_ = –(CH_2_)_6_NH_2_. The results represent mean values (± SD) from two independent experiments.

**Figure 3:**
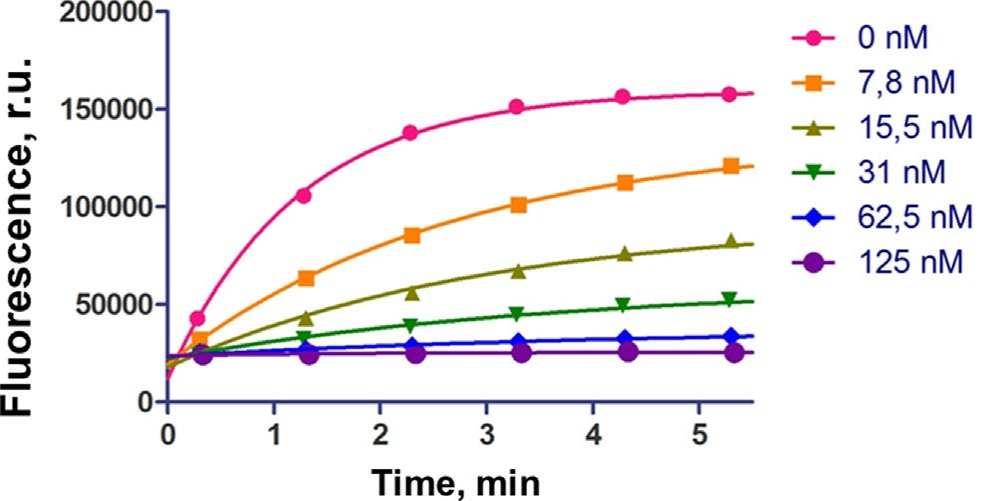
The influence of inhibiting oligoribonucleotide **r-inh** on hydrolysis of fluorescent RNA target. The cleavage conditions: 5 nM M1 RNA, 50 nM C5 protein, 100 nM fluorescent RNA target, 0–125 nM inhibiting oligonucleotide, 10 mM Tris-HCl, pH 7.5, 10 mM MgCl_2_, 100 mM NH_4_Cl, 37°C.

**Figure 3** shows the typical curves for hydrolysis of fluorescent RNA target upon inhibition of RNase P by **r-inh**. We observed the progressive decrease of enzyme activity upon the increase of the inhibitor concentration. The values of the half-maximal inhibitory concentration (IC_50_) were obtained using the equation (3) for competitive inhibition, by analogy with (Sabatino and Damha, 2007) (**Table 1**).

The non-modified oligoribonucleotide **r-inh** provided the maximal inhibitory effect. The change of ribose for 2’-*O*-methylated ribose (**m-inh**) or deoxyribose (**d-inh**) led to more modest inhibiting activity: the IC_50_ values increased approximately 10 times. There was no inhibiting effect in the case of uniformly modified phosphoryl guanidine oligonucleotide **pgd-inh1**. Nevertheless, selectively modified phosphoryl guanidine oligonucleotides **pgd-inh2** and **pgd-inh3** containing modified phosphodiester linkages in certain positions showed pronounced inhibiting activity. At that, **pgd-inh3** with phosphoryl guanidine-modified 3’-terminal fragment was more effective than **pgd-inh2** with the same modifications introduced close to the 5’-end. We chose two most effective modified oligonucleotide inhibitors **m-inh** (IC_50_ = 90 nM) and **pgd-inh3** (IC_50_ = 100 nM) for further studies. These oligomers combine RNase P inhibiting activity with good stability in biological media (Novopashina et al., 2018) (see **Figure S2**), so we consider them as a prospective basis for the development of antibacterial agents. Of note, the high level of structural and functional conservation of bacterial RNase P (Altman, 2011) permits to extrapolate the regularities obtained for *E.coli* enzyme to the other bacterial species, particularly *A. baumannii* (Davies-Sala et al., 2018).

Currently, several oligonucleotide constructions have been proposed as antibacterial agents (Woodford and Wareham, 2008; Cansizoglu and Toprak, 2017; Narenji et al., 2017; Równicki et al., 2018; Xue et al., 2018; González-Paredes et al., 2019). In this context, the pivotal challenges are the resistance of oligonucleotides to the nuclease digestion, the capability to penetrate bacterial cells, and the efficiency of interaction with a target bacterial molecule. To solve the problem of cell penetration, we coupled antibacterial oligonucleotide constructs with cell delivery agents (Good, 2003; Good and Stach, 2011; Giedyk et al., 2019). Four peptides were chosen as transporters: 19-mer fragment CM18 of cecropin-A/melittin hybrid peptide capable to disturb membrane (Salomone et al., 2013; Fasoli et al., 2014); 15-mer fragment of HGP peptide of gp41 HIV protein, which enhances endosomolytic activity (Kwon et al., 2008; Kwon et al., 2010); 16-mer oligohistidine peptide (Н_16_) (Iwasaki et al., 2015); and 17-mer analog of bombolytine V, membrane-destroying antimicrobial peptide (AMP), with all basic residues replaced by the glutamine acid (Ahmad et al., 2015).

**Figure 4:**
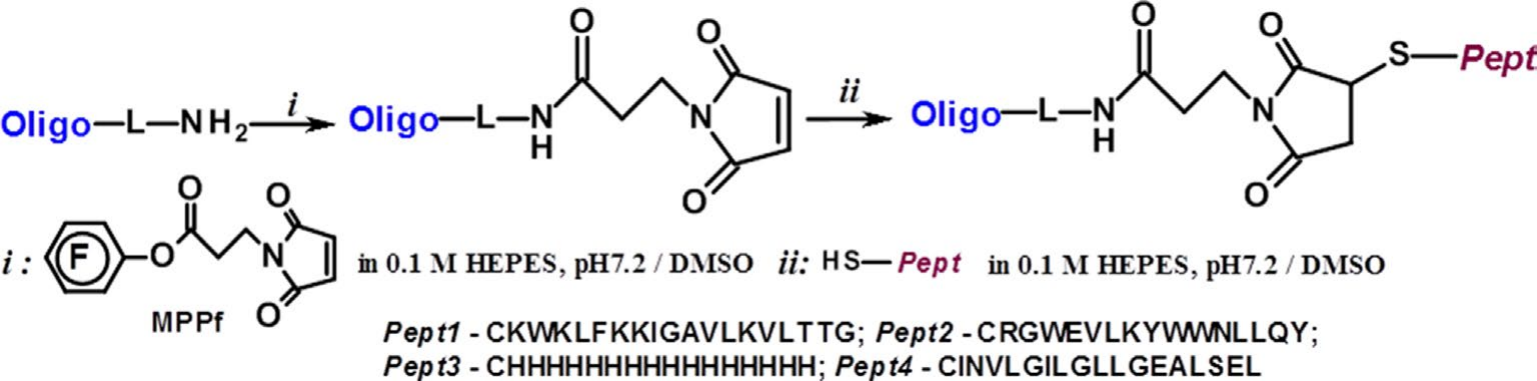
Scheme of the synthesis of the peptide conjugates of modified oligonucleotides **m-inh** and **pgd-inh**3.

For synthesizing oligonucleotide-peptide conjugates, we used the strategy based on thiol-maleimide conjugation chemistry (**Figure 4**). The maleimide group was attached to the 5’- or 3’-amino-modified oligonucleotide using MPPf synthesized by analogy with (Gruegelsiepe et al., 2003). The peptides containing N-terminal cysteine reacted with the maleimide-modified oligonucleotide, giving the covalent conjugates (**Table 2**). The degrees of the oligonucleotides’ conversion to the conjugates were about 70–90% by the HPLC data, depending on the type of the peptide. The conjugates were isolated by denaturating PAGE and analyzed by MALDI-TOF and ESI mass spectrometry (**Figure S7 and S8**).

**Table 2:** The peptide conjugates of RNase P inhibiting oligonucleotides **m-inh** and **pgd-inh3**.

No_	Code	Sequences 5’-3’	Molecular weight	
			Calc.	Found
**1**	**m-inh (control)**	5’-C^m^A^m^A^m^G^m^C^m^A^m^G^m^C^m^C^m^U^m^A^m^C^m^C^m^C^m^-3’	4,763.2	4,765.1^1^*
**2**	**5’-** ***Pept2*** **-m-inh-** ***Flu***	5’-***Pept2***-C^m^A^m^A^m^G^m^C^m^A^m^G^m^C^m^C^m^U^m^A^m^C^m^C^m^C^m^-***Flu***-3’	7,582.4	7,578.8^1^*
**3**	**5’-** ***Pept3*** **-m-inh-** ***Flu***	5’-***Pept3***-C^m^A^m^A^m^G^m^C^m^A^m^G^m^C^m^C^m^U^m^A^m^C^m^C^m^C^m^-***Flu***-3’	7,740.3	–
**4**	**5’-** ***Pept4*** **-m-inh-** ***Flu***	5’-***Pept4***-C^m^A^m^A^m^G^m^C^m^A^m^G^m^C^m^C^m^U^m^A^m^C^m^C^m^C^m^-***Flu***-3’	7,252.1	–
**5**	**m-inh-** ***Flu***	5’-C^m^A^m^A^m^G^m^C^m^A^m^G^m^C^m^C^m^U^m^A^m^C^m^C^m^C^m^ –***Flu***-3’	5,152.6	5,153.4^1^*
**6**	**5’-** ***Pept2*** **-m-inh**	5’-***Pept2***-C^m^A^m^A^m^G^m^C^m^A^m^G^m^C^m^C^m^U^m^A^m^C^m^C^m^C^m^–3’	7,095.9	7,095.9^1^*
**7**	**5’-** ***Pept3*** **-m-inh**	5’-***Pept3***-C^m^A^m^A^m^G^m^C^m^A^m^G^m^C^m^C^m^U^m^A^m^C^m^C^m^C^m^–3’	7,253.8	7,252.1^1^*
**8**	**5’-** ***Pept4*** **-m-inh**	5’-***Pept4***-C^m^A^m^A^m^G^m^C^m^A^m^G^m^C^m^C^m^U^m^A^m^C^m^C^m^C^m^-3’	6,765.6	6,762.5^1^*
**9**	**3’-** ***Pept2*** **-m-inh**	5’-C^m^A^m^A^m^G^m^C^m^A^m^G^m^C^m^C^m^U^m^A^m^C^m^C^m^C^m^-***Pept2***-3’	7,095.9	–
**10**	**3’-** ***Pept3*** **-m-inh**	5’-C^m^A^m^A^m^G^m^C^m^A^m^G^m^C^m^C^m^U^m^A^m^C^m^C^m^C^m^-***Pept3***-3’	7,253.8	7,252.4^1^*
**11**	**3’-** ***Pept4*** **-m-inh**	5’-C^m^A^m^A^m^G^m^C^m^A^m^G^m^C^m^C^m^U^m^A^m^C^m^C^m^C^m^-***Pept4***-3’	6,765.6	6,763.5^1^*
**12**	**pgd-inh3**	5’-d(CAAGCAGCxCxTxAxCxCxC)-3’	4,933.8	4,936.1^2^*
**13**	**5’-** ***Pept1*** **-pgd-inh3-** ***Flu***	5’-***Pept1***-d(CAAGCAGCxCxTxAxCxCxC)–***Flu***-3’	7,907.3	7,911.2^2^*
**14**	**5’-** ***Pept2*** **-pgd-inh3-** ***Flu***	5’-***Pept2***-d(CAAGCAGCxCxTxAxCxCxC)–***Flu***-3’	7,752.9	7,748.0^2^*
**16**	**5’-** ***Pept3*** **-pgd-inh3-** ***Flu***	5’-***Pept3***-d(CAAGCAGCxCxTxAxCxCxC)–***Flu***-3’	7,910.8	7,904.5^2^*
**17**	**5’-** ***Pept4*** **-pgd-inh3-** ***Flu***	5’-***Pept4***-d(CAAGCAGCxCxTxAxCxCxC)–***Flu***-3’	7,601.2	7,597.9^2^*
**18**	**pgd-inh-Flu**	5’-d(CAAGCAGCxCxTxAxCxCxC)-***Flu***-3’	5,598.4	5,586.7^2^*
**19**	**5’-** ***Pept2*** **-pgd-inh3**	5’-***Pept2***-d(CAAGCAGCxCxTxAxCxCxC)-3’	7,346.4	7,341.3^2^*
**20**	**5’-** ***Pept3*** **-pgd-inh3**	5’-***Pept3***-d(CAAGCAGCxCxTxAxCxCxC)-3’	7,503.9	–
**21**	**5’-** ***Pept4*** **-pgd-inh3**	5’-***Pept4***-d(CAAGCAGCxCxTxAxCxCxC)-3’	7,016.1	–
**22**	**3’-** ***Pept1*** **-pgd-inh3**	5’-d(CAAGCAGCxCxTxAxCxCxC)-***Pept1***-3’	7,338.6	7,330.1^2^*
**23**	**3’-** ***Pept2*** **-pgd-inh3**	5’-d(CAAGCAGCxCxTxAxCxCxC)-***Pept2***-3’	7,346.4	–

N^m^ – A^m^,U^m^,C^m^,G^m^ – 2’-O-methylribonucleotides, Nx – phosphoryl guanidine deoxyribonucleotide, Flu – fluorescein residue, **Pept1**– CKWKLFKKIGAVLKVLTTG, **Pept2** – CRGWEVLKYWWNLLQY, **Pept3** – CHHHHHHHHHHHHHHHH, **Pept4** – CINVLGILGLLGEALSEL. ^1^* – Determined by MALDI-TOF mass-spectrometry. ^2^* – Determined by ESI mass-spectrometry.

**Figure 5:**
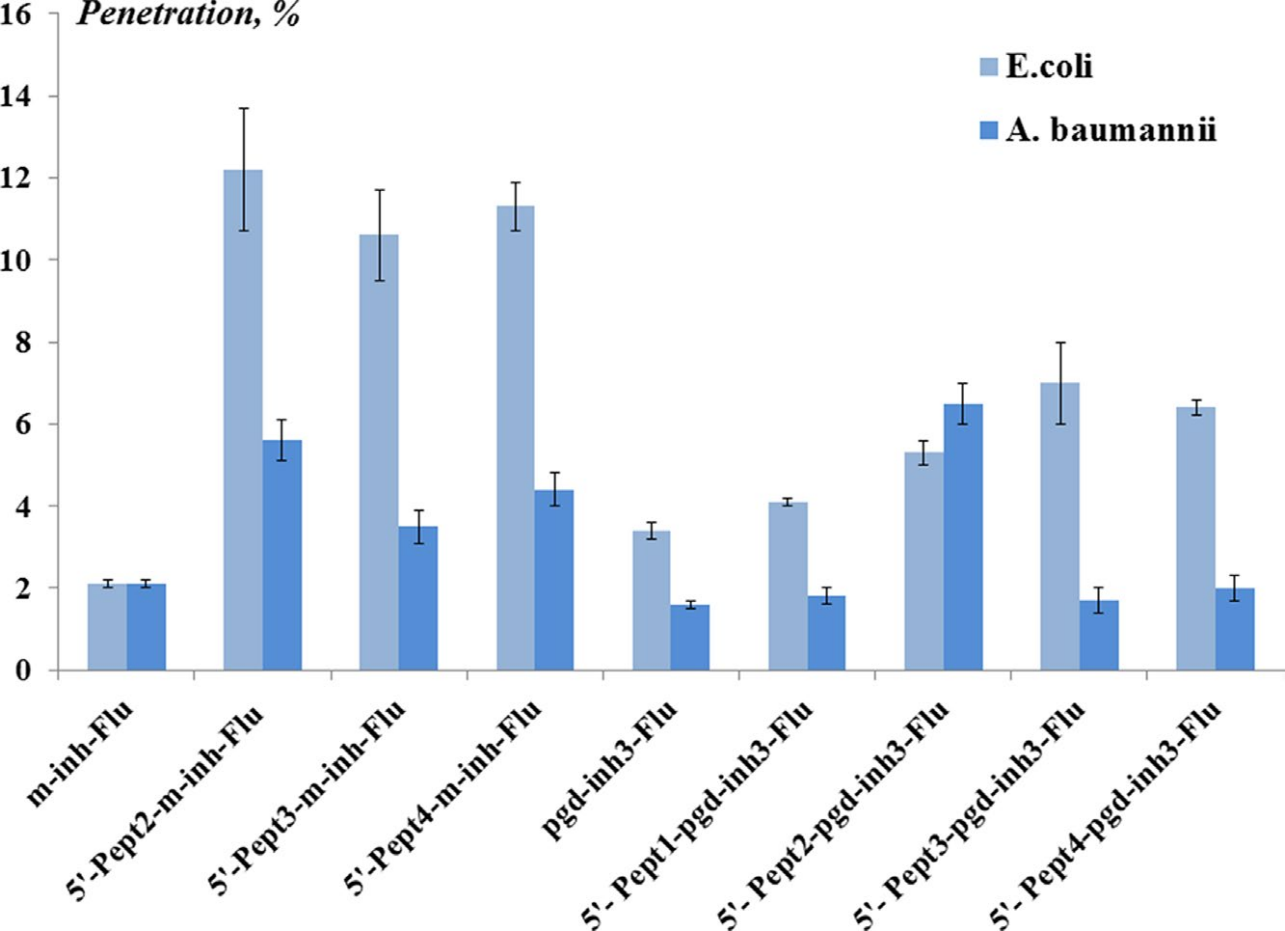
The level (%) of oligonucleotide conjugates penetration in *Escherichia coli* and *Acinetobacter baumannii* obtained by flow cytometry. The concentration of the conjugates was 1 µM. The cells were incubated with conjugates for 1 h at 37°C. The results are mean values (± SD) from four independent experiments. The fluorescence of the sample of bacterial cells incubated with fluorescein-labeled peptide conjugate of the oligonucleotide is taken as 100%.

For cell penetration studies, we employed 5’-peptide conjugates of oligonucleotides bearing the 3’-fluorescein label. The levels of penetration in *E. coli* and *A. baumannii* were estimated using flow cytometry (**Figure 5**, **Table S2**), and the intracellular distribution of conjugates was visualized using confocal microscopy (**Figure S3, S4 and Table S3**).

Fluorescent oligo(2’-O-methylribonucleotide) **m-inh-Flu** penetrated *E. coli* cells at the level of 2%. However, the levels of cell penetration were significantly higher for peptide conjugates of **m-inh-Flu**. Approx. 10–12% of oligonucleotides, depending on the peptide, were found in *E. coli*. Meanwhile, the levels of penetration in *A. baumannii* were approx. 3–6%. Peptide conjugates of phosphoryl guanidine oligonucleotide **pgd-inh3-Flu** demonstrated fewer levels of penetration into the bacterial cells. The best result was observed for ***5’-Pept2*** **-pgd-inh3-** ***Flu***, which penetrated *A. baumannii* at the level of 6.5%.

The obtained results indicated that conjugation with peptides facilitates the penetration of modified oligonucleotides into bacterial cells of both types. Nearly in all cases, we observed better penetration for *E. coli* cells compared to *A. baumannii*. This phenomenon might be explained by distinct differences either in the cell wall structure or in efflux systems of these two gram-negative bacteria. Therefore, the approach to intracellular delivery requires optimization when passing from one bacterium to another, even within the same class.

**Figure 6:**
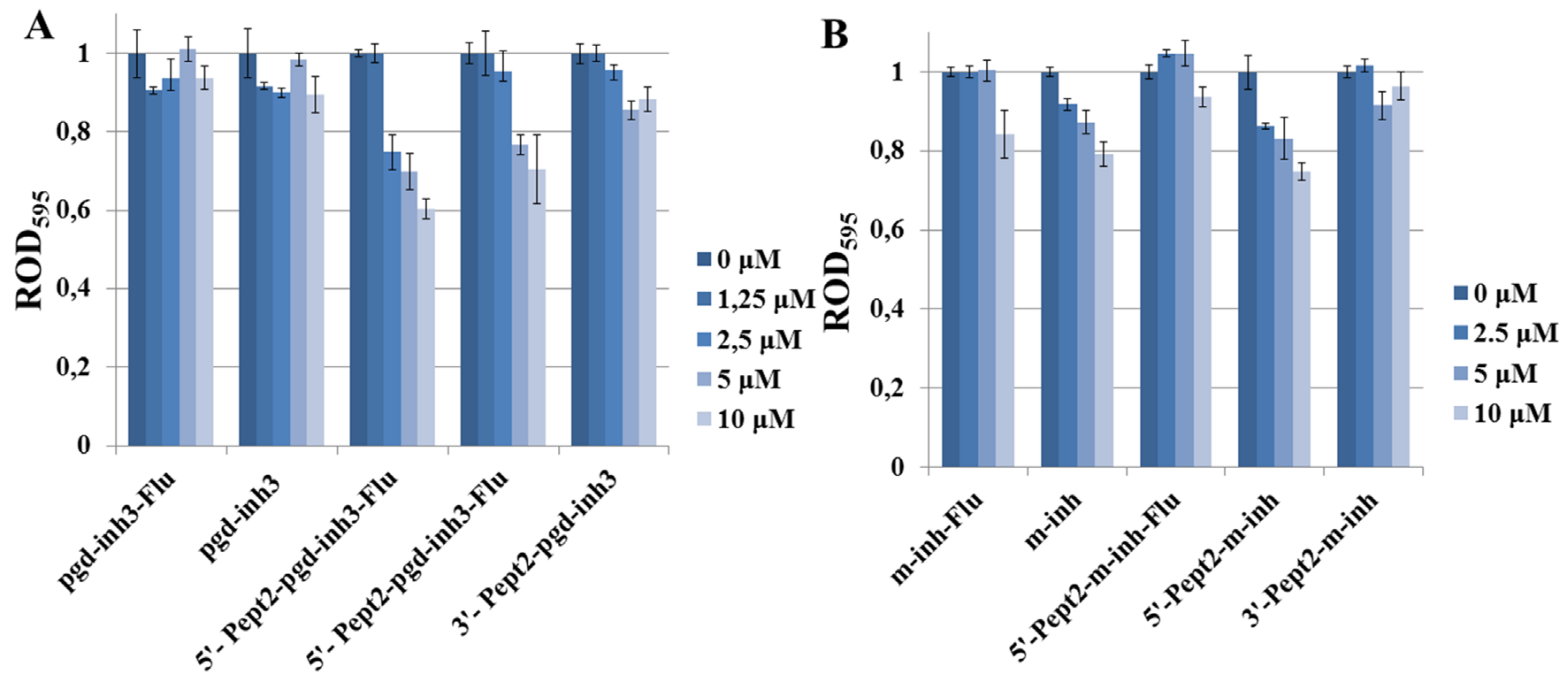
Influence of the peptide conjugates of inhibiting phosphoryl guanidine oligonucleotide **(A)** and oligo(2’-O-methylribonucleotide) **(B)** on *E. coli* growth. Concentrations of conjugates in the media were 0–10 µM. Bacterial cells were incubated in the cell culture plate (96-well) at 37°C and 580 rpm during 22 h. ROD_595_—relative optical density, the ratio of OD_495_ for the sample and oligonucleotide-free control. Values are mean ± SD (n = 3).

The oligonucleotides and their conjugates were tested on their antibacterial properties using *E. coli* as a target. We used approximately 60 mg/ml concentrations of oligonucleotide inhibitors, which are comparable with the minimum inhibiting concentration (MIC) for standard antibiotics (50–300 mg/ml) (Wannigama et al., 2019). Namely, we studied conjugates of **m-inh** and **pgd-inh3** with ***Pept2*** peptide at the 5’-end, either with or without 3’-fluorescein residue (**Figure 6**), and the same oligonucleotides with Pept2 at the 3’-end. Despite the relatively low level of the cell penetration for phosphoryl guanidine oligonucleotide, we observed the suppression of *E. coli* growth for 5’-peptide conjugates, and the presence of fluorescein residue enhanced the effect to some extent (**Figure 6A**). Inhibiting 2’-OMe RNA oligonucleotides suppressed the bacterial growth irrespective of the absence or presence of 5’-peptide. Upon that, although their level of cell penetration was higher than that for phosphoryl guanidines, the inhibiting effect was somewhat lower as compared to peptide conjugates of **pgd-inh3** (**Figure 6B**). For both types of modified oligonucleotides, peptide attached to the 3’-end had no impact on their inhibiting activities: unconjugated oligomers and their 3’-peptide conjugates suppressed the bacterial growth to the same extent. We suppose that the presence of bulk peptide fragment at the 3’-end causes steric hindrance for binding of 3’-CCA fragment to M1RNA in P15 loop site. We also observed all abovementioned regularities for conjugates with the peptides ***Pept1***, ***Pept3***, and ***Pept4*** (see **Figure S5 and S6**).

Enhancement of antibacterial effect upon attachment of peptides to the 5’-end of modified oligonucleotide inhibitors of RNase P proves the feasibility of the proposed approach. We had not observed any correlation between the level of cell penetration of the oligonucleotide and their ability to suppress the bacterial growth. Peptide conjugates of 2’-OMe RNA demonstrated relatively good cell penetration, but rather low antibacterial activity. Relevant conjugates of the phosphoryl guanidine oligonucleotide were less effective in cell penetration but showed better results in suppression of bacterial growth. We hypothesize that the optimization of cell-penetrating properties of phosphoryl guanidine oligonucleotides would improve their antibacterial properties. Further studies are required to prove this suggestion, directed to revealing the roles of oligonucleotide and peptide counterparts in the cell penetration and growth suppression. With this knowledge, we would be able to optimize the structure of oligonucleotide-peptide conjugates inhibiting RNase P to improve their antibacterial activity.

## Data Availability

All datasets generated for this study are included in the manuscript and/or the supplementary files.

## Author Contributions

DN and MV conceived and designed the experiments. AN, AD, ND, LK, AM, AB, and MK performed the experiments. NT, DP, SA, and AV analyzed the data and co-wrote the paper.

## Funding

The research was carried out with financial support by the RFBR grant N 17-04-01892. In the part of the synthesis of modified oligonucleotides, the work was supported by the Russian State-funded budget project of ICBFM SB RAS # AAAA-A17-117020210021-7.

## Conflict of Interest Statement

The authors declare that the research was conducted in the absence of any commercial or financial relationships that could be construed as a potential conflict of interest.
