# Supplementary material for: Novel Peptide Conjugates of Modified Oligonucleotides for Inhibition of Bacterial RNase P
Source: Front Pharmacol. 2019 Jul 19;10:813. doi: 10.3389/fphar.2019.00813 (PMC6658616; doi:10.3389/fphar.2019.00813)
Supplement: Supplementary file 1 [file DataSheet_1.pdf]

Supplementary Material

1 Assessment of the catalytic activity of RNase P on the model fluorescent RNA target

RNA substrate: 5'-**Flu**-pGUUUUCUUCGGUGGGGUUUCUUCCCCACCACCA-**BHQ1**-3'

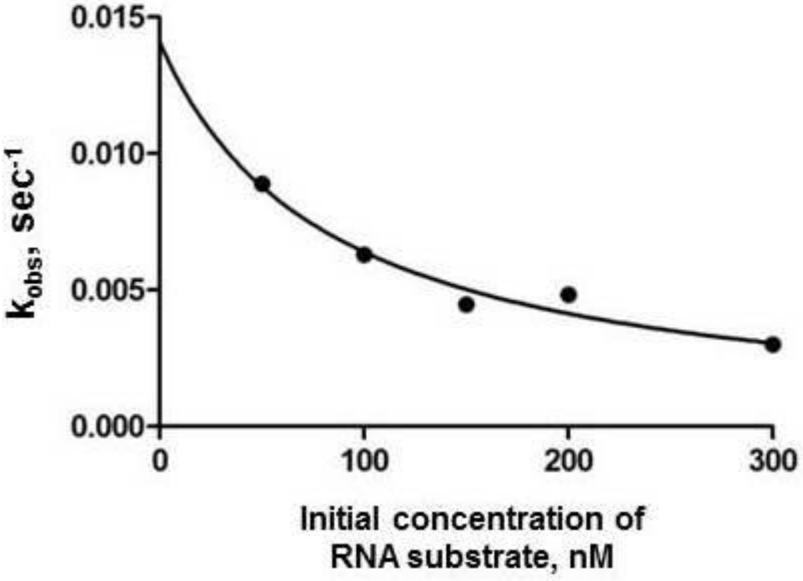

Supplementary Figure S1. Concentration dependence of k_{obs} values for hydrolysis of fluorescent RNA target.

Table S1. Calculated kinetic parameters of fluorescent RNA target hydrolysis by RNase P

**Parameter**	**Value**
Michaelis constant (K_m)	83 ± 49 nM
The maximum reaction rate (V_{max})	120 ± 24 nM·min^{-1}
Turnover number (k_{cat})	24 ± 5 min^{-1}

2 Stability of inhibiting oligonucleotides in culture media

Stability of the inhibiting oligonucleotides to the nucleases digestion was investigated in the model system using DMEM culture medium (Life Technologies, USA) containing 10% fetal bovine serum (FBS, heat inactivated, Invitrogen, USA). The treatment of 5'-[^{32}P]-labeled oligonucleotides (**r-inh**, **m-inh**, **d-inh**) by 10% FBS in DMEM was carried out at 37°C. The 5 µl aliquots were taken after 15, 30,60, 120, 240, 360 minutes and one day, mixed with the Stop-mix solution and analyzed by denaturating 15% PAGE (acrylamide–N,N'-methylenebisacrylamide 30 : 1, 8 M urea, 50 mM Tris-borate (pH 8.3), 0.1 mM Na_2EDTA) followed by radioautography. The percentage of initial

oligonucleotide in the reaction mixture was calculated from the autoradiography data as the ratio of the intensity of the band corresponding to the initial oligonucleotide to the total intensity in the line (Fig. S2).

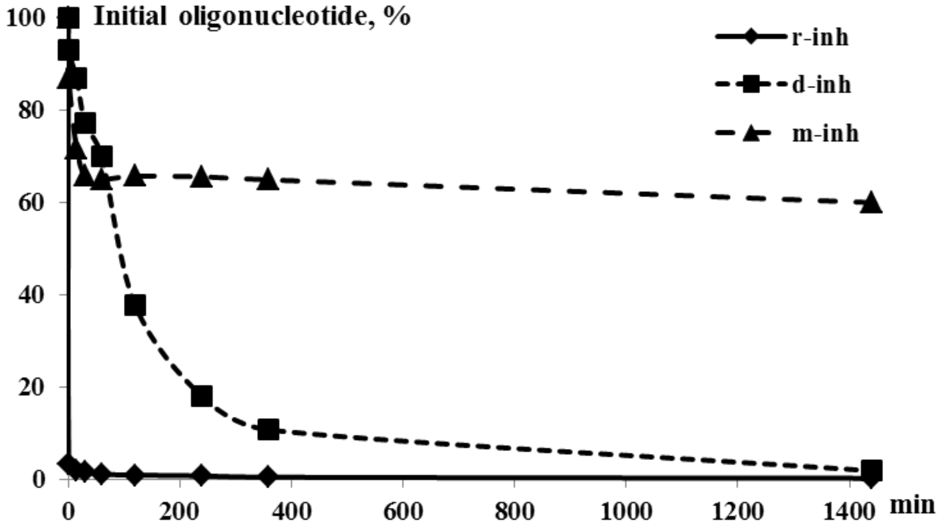

Supplementary Figure S2. The hydrolysis of inhibiting oligonucleotides **r-inh**, **d-inh**, **m-inh** in the presence of DMEM containing 10% FBS at 37 °C.

The inhibiting oligoribonucleotide **r-inh** was entirely digested by nucleases immediately after the addition of DMEM with FBS. The inhibiting oligodeoxyribonucleotide **d-inh** degraded for 50% after 2 h of incubation. The most stable inhibiting oligo(2'-O-methylribonucleotide) **m-inh** was cleaved for 40% during one day of incubation.

The stability of phosphorylguanidine oligonucleotides was investigated and published earlier [19]. The data confirm that phosphorylguanidine oligonucleotides are completely stable in 50% FBS up to 3 weeks.

3 Fluorescently labeled inhibiting oligonucleotides and their conjugates with peptides

Fluorescein-modified CPG carrier (Glen Research, USA) was used for phosphoramidite oligonucleotide synthesis of fluorescein-labeled oligo(2'-O-methylribonucleotide) and phosphorylguanidine oligonucleotide. 5'-Aminolink (Lumiprobe, Russia) was used to introduce 5'-$NH_2(CH_2)_6$- group to the oligonucleotides. Conjugation of 3'-fluorescein-labeled 5'-amino modified oligonucleotides with peptides was performed as described in Materials and Methods. The synthesized conjugates are listed in Table S2.

Table S2. Fluorescently labeled oligonucleotides inhibitors of RNase P and their peptide conjugates.

**Code**	**Sequence 5'-3'**
**m-inh-*Flu***	5'-$C^mA^mA^mG^mC^mA^mG^mC^mC^mU^mA^mC^mC^mC^m$ –***Flu***-3'
Pept2*-m-inh-*Flu	5'-***Pept2***-$C^mA^mA^mG^mC^mA^mG^mC^mC^mU^mA^mC^mC^mC^m$ –***Flu***-3'

Pept3*-m-inh-*Flu	5'-***Pept3***-$C^mA^mA^mG^mC^mA^mG^mC^mC^mU^mA^mC^mC^mC^m$ –***Flu***-3'
Pept4*-m-inh-*Flu	5'-***Pept4***-$C^mA^mA^mG^mC^mA^mG^mC^mC^mU^mA^mC^mC^mC^m$ –***Flu***-3'
**pdg-inh3-*Flu***	5'-d(CAAGCAGCxCxTxAxCxCxC)–***Flu***-3'
Pept1*-pgd-inh3-*Flu	5'-***Pept1***-d(CAAGCAGCxCxTxAxCxCxC)–***Flu***-3'
Pept2*-pgd-inh3-*Flu	5'-***Pept2***-d(CAAGCAGCxCxTxAxCxCxC)–***Flu***-3'
Pept3*-pgd-inh3-*Flu	5'-***Pept3***-d(CAAGCAGCxCxTxAxCxCxC)–***Flu***-3'
Pept4*-pgd-inh3-*Flu	5'-***Pept4***-d(CAAGCAGCxCxTxAxCxCxC)–***Flu***-3'

Pept1 – CKWKLFKKIGAVLKVLTTG; ***Pept2*** – CRGWEVLKYWWNLLQY; ***Pept3*** – CHHHHHHHHHHHHHHHH; ***Pept4*** – CINVLGILGLLGEALSEL; ***Flu*** – fluorescein; N^m – 2'-O-methylribonucleotide; Nx – phophorylguanidine deoxyribonucleotide; x - phosphate groups modified with 1,3-dimethylimidazolidine-2-imine residues.

4 The penetration of peptide conjugates into bacterial cells

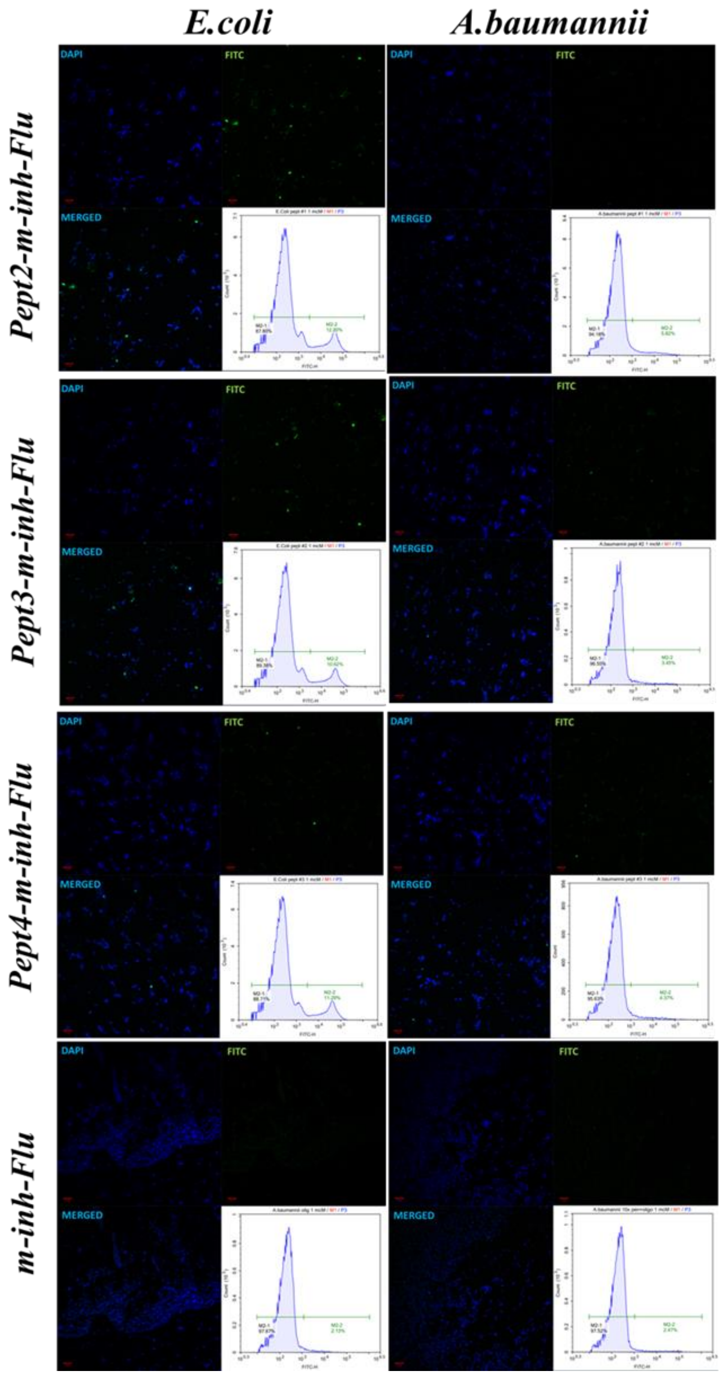

Supplementary Figure S3. Confocal microscopy and cytometry analysis of **m-inh** peptide conjugates' penetration into bacterial cells. The concentration of conjugates was 1 μM.

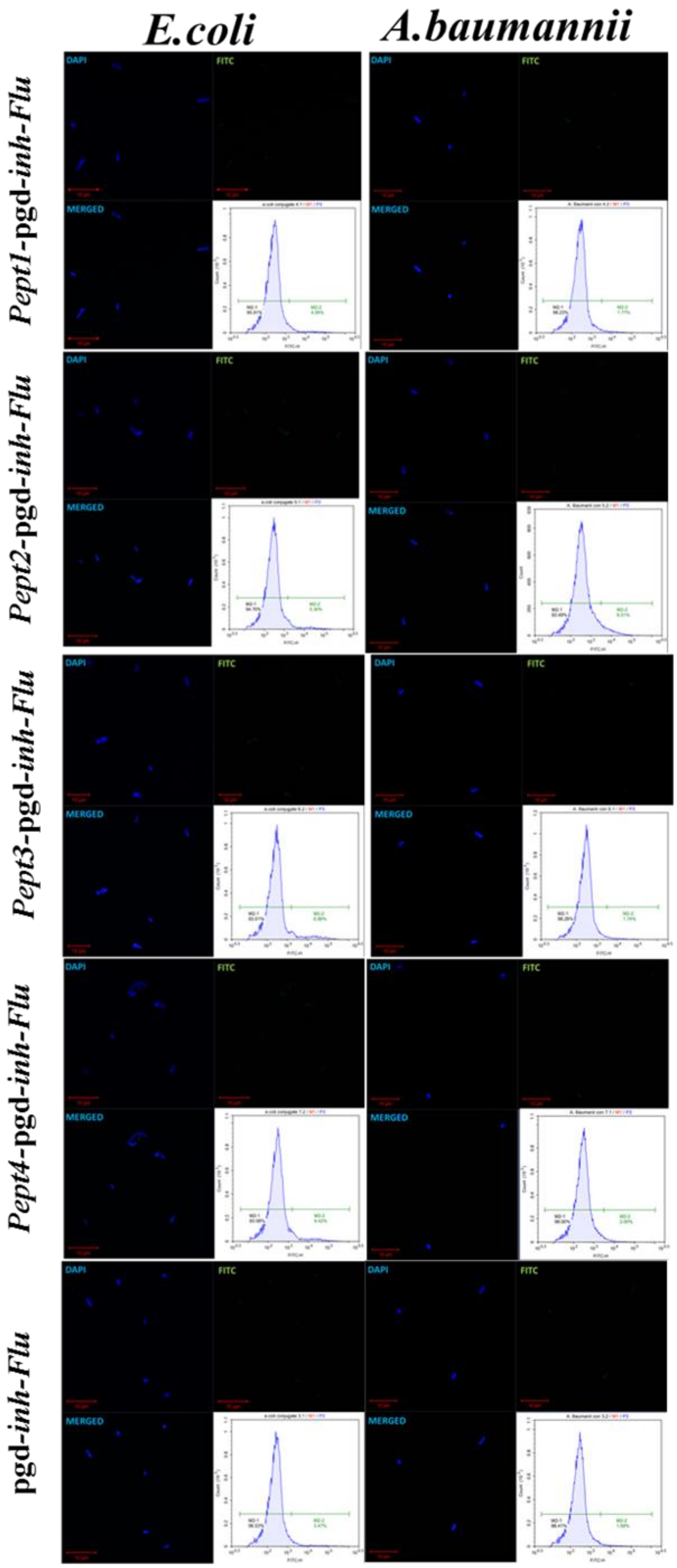

Supplementary Figure S4. Confocal microscopy and cytometry analysis of **pgd-inh** peptide conjugates' penetration into bacterial cells. The concentration of conjugates was 1 μM.

Table S3. The level of peptide conjugates' penetration into bacterial cells

**Code**	***Level of penetration, %****	
	E.coli	***A. baumannii***
**m-inh-*Flu***	2,1±0,1	2,1±0,1
**5'-*Pept2*-m-inh-*Flu***	12,2±1,5	5,6±0,5
**5'-*Pept3*-m-inh-*Flu***	10,6±1,1	3,5±0,4
**5'-*Pept4*-m-inh-*Flu***	11,3±0,6	4,4±0,4
**pgd-inh3-*Flu***	3,4±0,2	1,6±0,1
5'- Pept1*-pgd-inh3-*Flu	4,1±0,1	1,8±0,2
5'- Pept2*-pgd-inh3-*Flu	5,3±0,3	6,5±0,5
5'- Pept3*-pgd-inh3-*Flu	7,0±1	1,7±0,3
5'- Pept4*-pgd-inh3-*Flu	6,4±0,2	2,0±0,3

* data obtained by cytometry method. The results are mean values (± SD) from four independent experiments.

5 The suppression of the bacterial growth

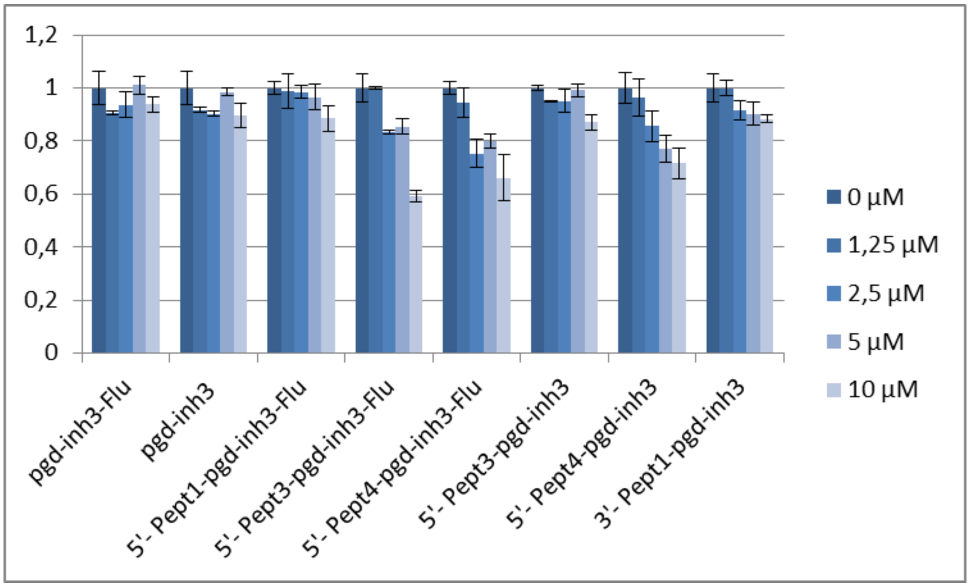

Supplementary Figure S5. Bacterial cells (*E. coli* (DH5α)) were incubated in cell culture plate (96-well) at 37°C and 580 rpm during 22 h. The culture growth was estimated by optical density in each well. Value 1.0 corresponds to the control bacteria incubated without peptide conjugates or oligonucleotides. Values are mean ± SD (n=3).

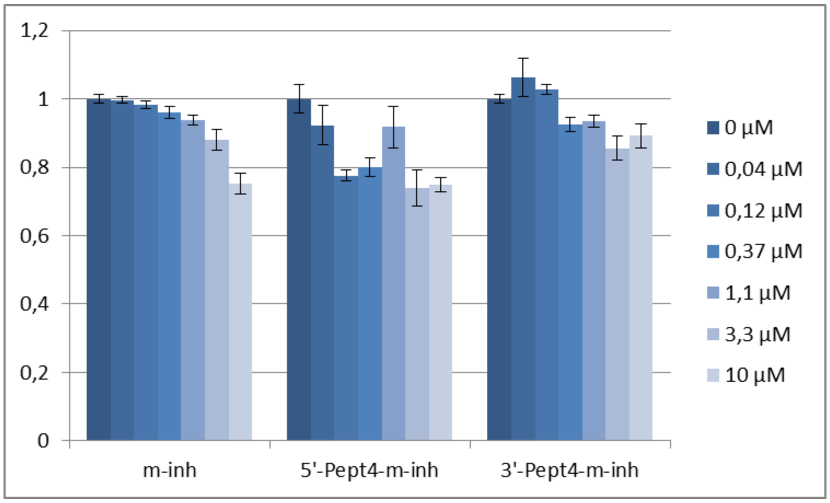

Supplementary Figure S6. Bacterial cells (*E.coli* (DH5α)) were incubated in cell culture plate (96-well) at 37°C and 580 rpm during 22 h. The culture growth was estimated by optical density in each well. Value 1.0 corresponds to the control bacteria incubated without peptide conjugates or oligonucleotides. Values are mean ± SD (n=3).

6 Isolation of peptide conjugates

The reaction mixtures after synthesis of peptide conjugates were analyzed by Reverse Phase-HPLC (RP-HPLC) (Fig. S7) and by electrophoresis in 15% PAAG (Fig. S8). RP-HPLC analysis of the oligonucleotides and their conjugates is performed on an Alphachrome (Bioset, Russia) high performance liquid chromatography with the use of a ProntoSil-120-5-C18 AQ (75×2.0 mm, 5.0 µm) column, applying a gradient elution from 0 to 50% (15 min) of acetonitrile in 0.02 M triethylammonium acetate buffer, pH 7.0 at a flow rate 100 µL per min, and detection at 260 nm.

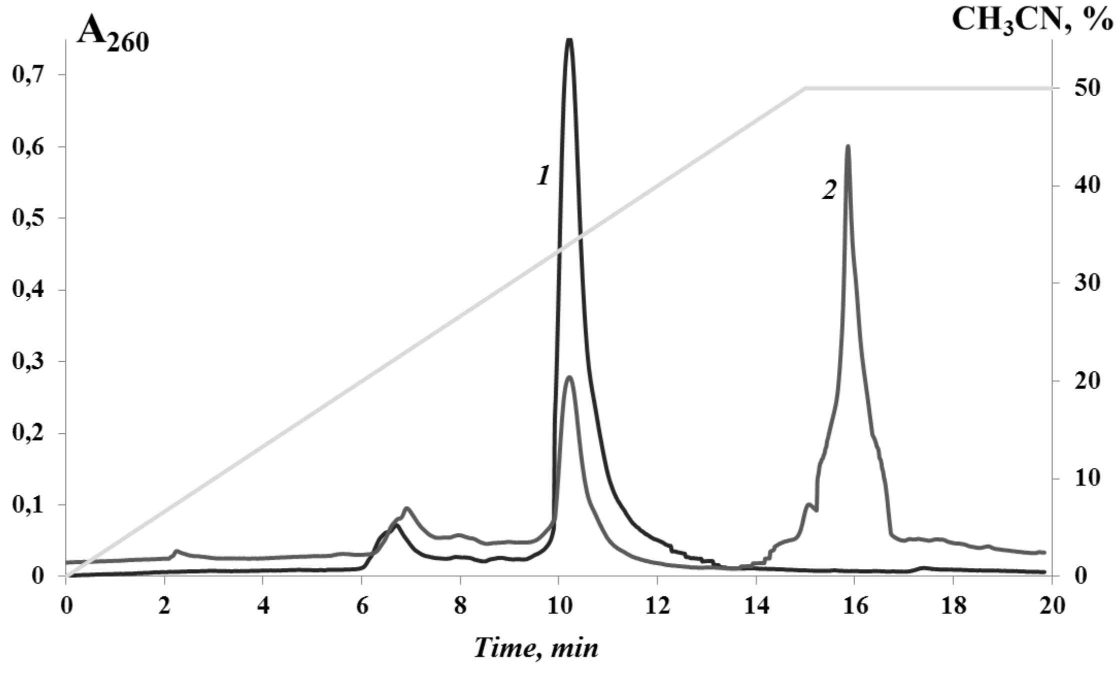

Supplementary Figure S7. HPLC analysis of initial (***curve 1***) 5'-aminomodified phosphorylguanidine oligonucleotide 5'-NH_2-$(CH_2)_6$-p-d(CAAGCAGCxCxTxAxCxCxC) and reaction mixture upon 5'-peptide conjugate 5'-***Pept2***-d(CAAGCAGCxCxTxAxCxCxC) preparation (***curve 2***).

For purification of the peptide conjugate 15-20 OU_{260} of the reaction mixture in the loading buffer was applied in the 3 cm width well of a 12% denaturing PAAG slab with the thickness of 1 mm. The products were detected by a UV-shadowing method. The product bands were excised and extracted by soaking the gel slice in 0.3 M $NaClO_4$, then isolate the conjugate by desalting using centrifugal filters Amicon Ultra (Merck Millipore, Germany) with subsequent precipitation with by 2% sodium perchlorate solution in acetone. For the analysis 0.05 OU_{260} of the reaction mixture or isolated peptide conjugate in the loading buffer were applied in the well of a 15% denaturing PAAG slab. The oligonucleotides were analyzed by gel staining using the solution of "Stains-all" dye. The products are visualized as violet or blue bands of lower mobility related to the control initial oligonucleotides (**Fig.S8**).

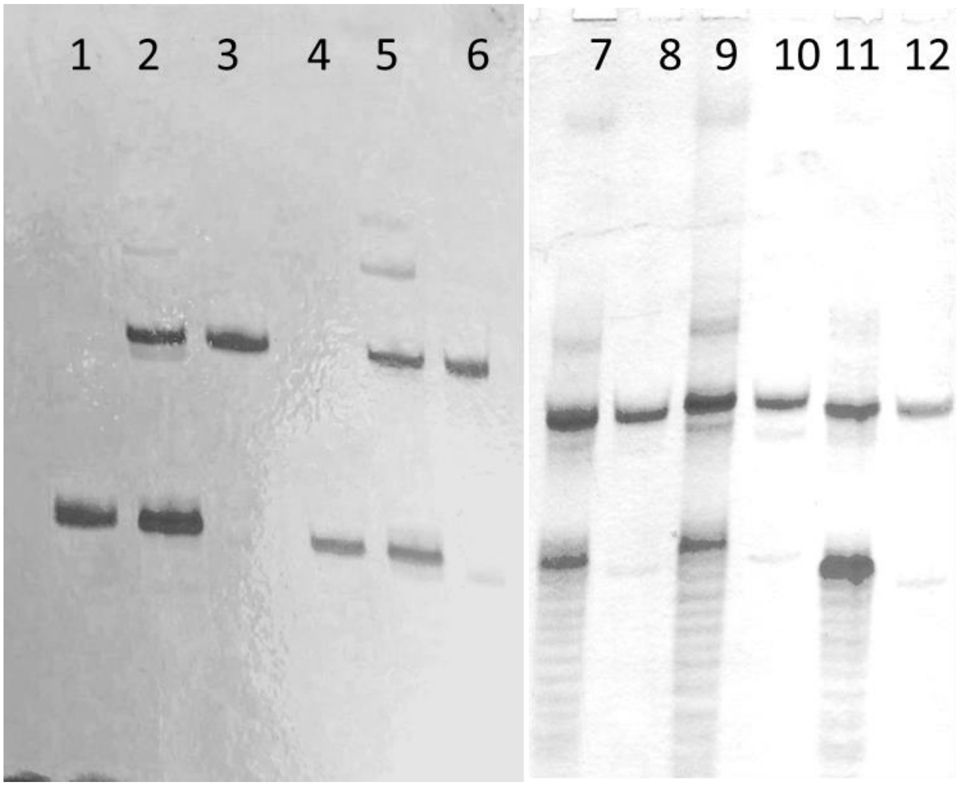

Supplementary Figure S8. Electrophoretic analysis of the reaction mixture (lines **2, 5, 7, 9, 11**) and peptide conjugates isolated by preparative PAAG electrophoresis (lines **3, 6, 8,10, 12**). Lines 1 and 4 – initial amino-modified oligonucleotides. Conditions: 15% denaturating PAAG (acrylamide/N,N'-methylene bis-acrylamide (19:1) containing 7 M urea in TBE buffer: 0.089M Tris–H3BO3, 0.001M Na2EDTA, pH 8.3). Lines (**1-3**) **- 5'-*Pept3*-m-inh,** lines (**4-6**) **- 3'-*Pept3*-m-inh,** lines (**7,8**) **- 5'-*Pept2*-pgd-inh3,** lines (**9,10**) **- 5'-*Pept3*-pgd-inh3,** lines (**11,12**) **- *3'-Pept2*-pgd-inh3.**

7 MS analysis of peptide conjugates

The peptide conjugates were analyzed by MALDI-TOF and ESI mass spectrometry. The mass spectra of the oligo(2'-O-methylribonucleotide) conjugates were recorded on a MALDI-TOF Autoflex Speed mass-spectrometer (Bruker Daltonics, Germany). The mass spectra of the phosphorylguanidine oligonucleotides conjugates were obtained using ESI-MS on the Agilent G6410A LC-MS/MS instrument (Agilent Technologies, USA).

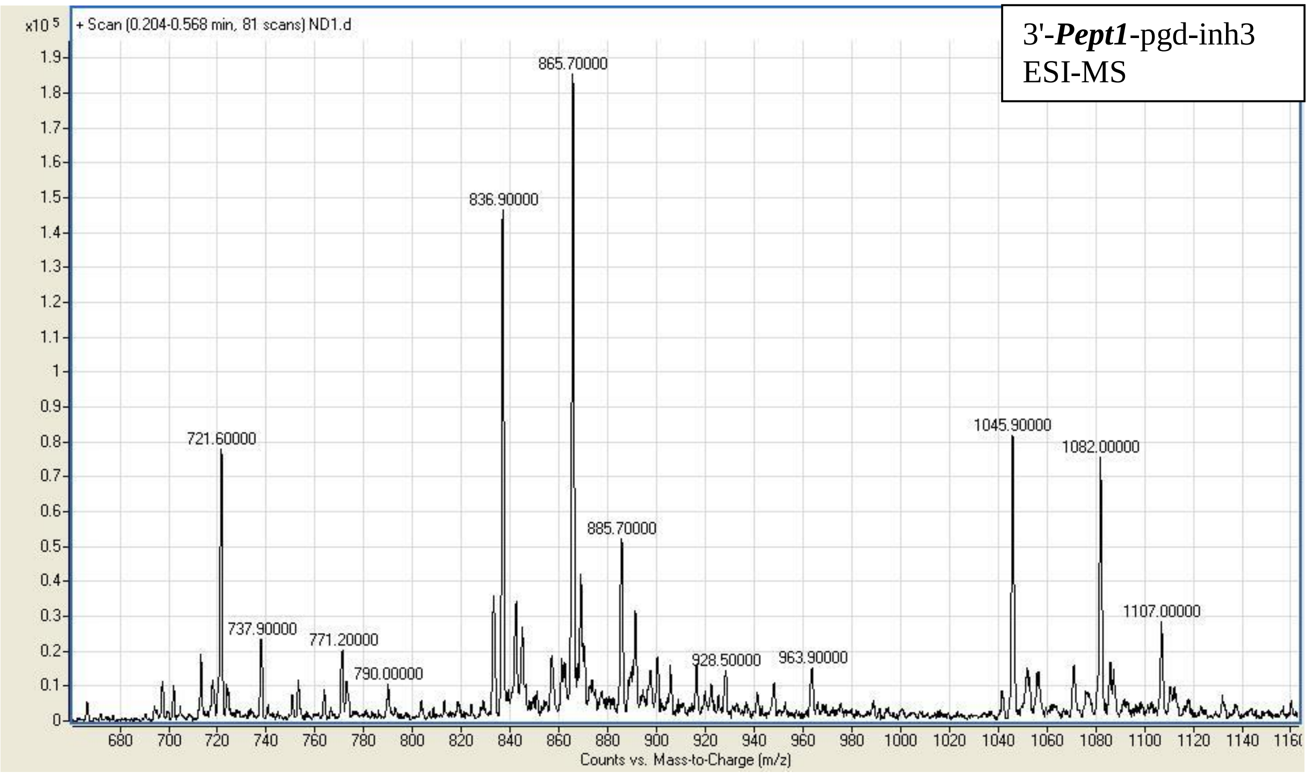

3'-***Pept1***-pgd-inh3
ESI-MS

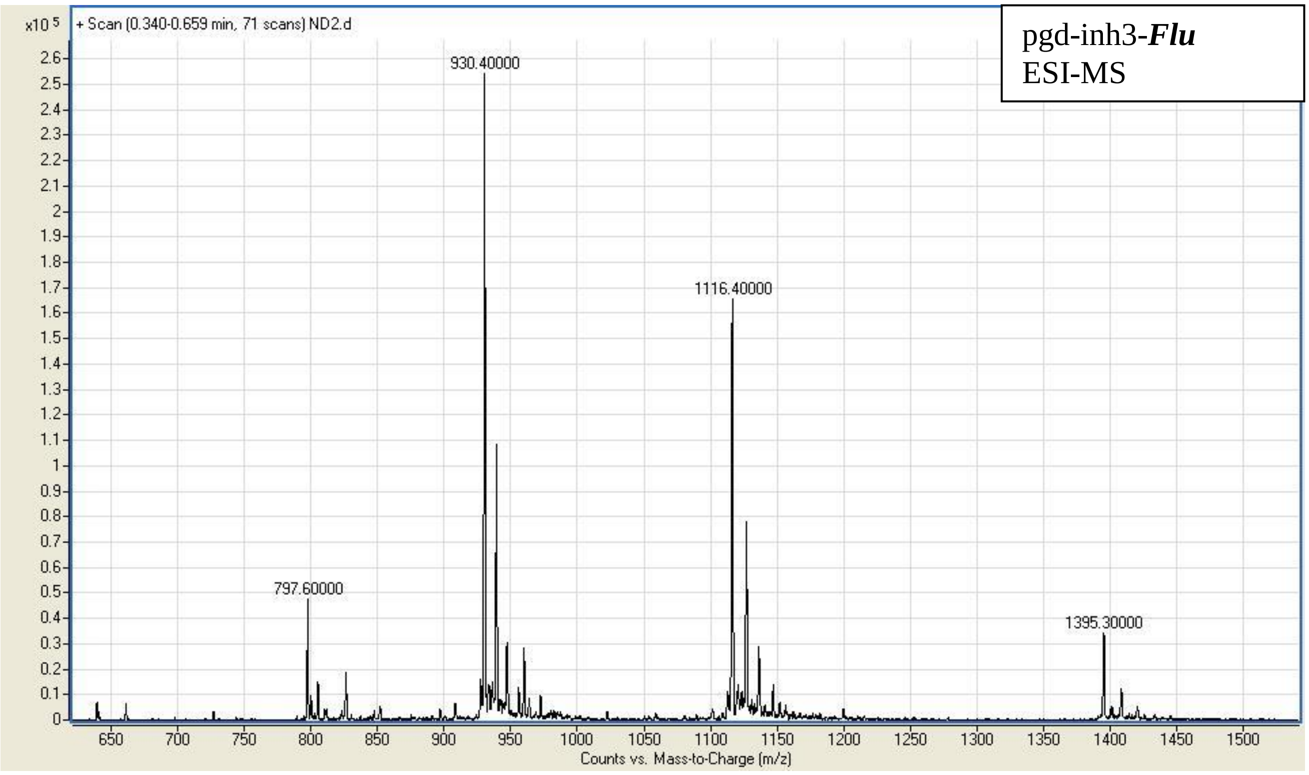

pgd-inh3-***Flu***
ESI-MS

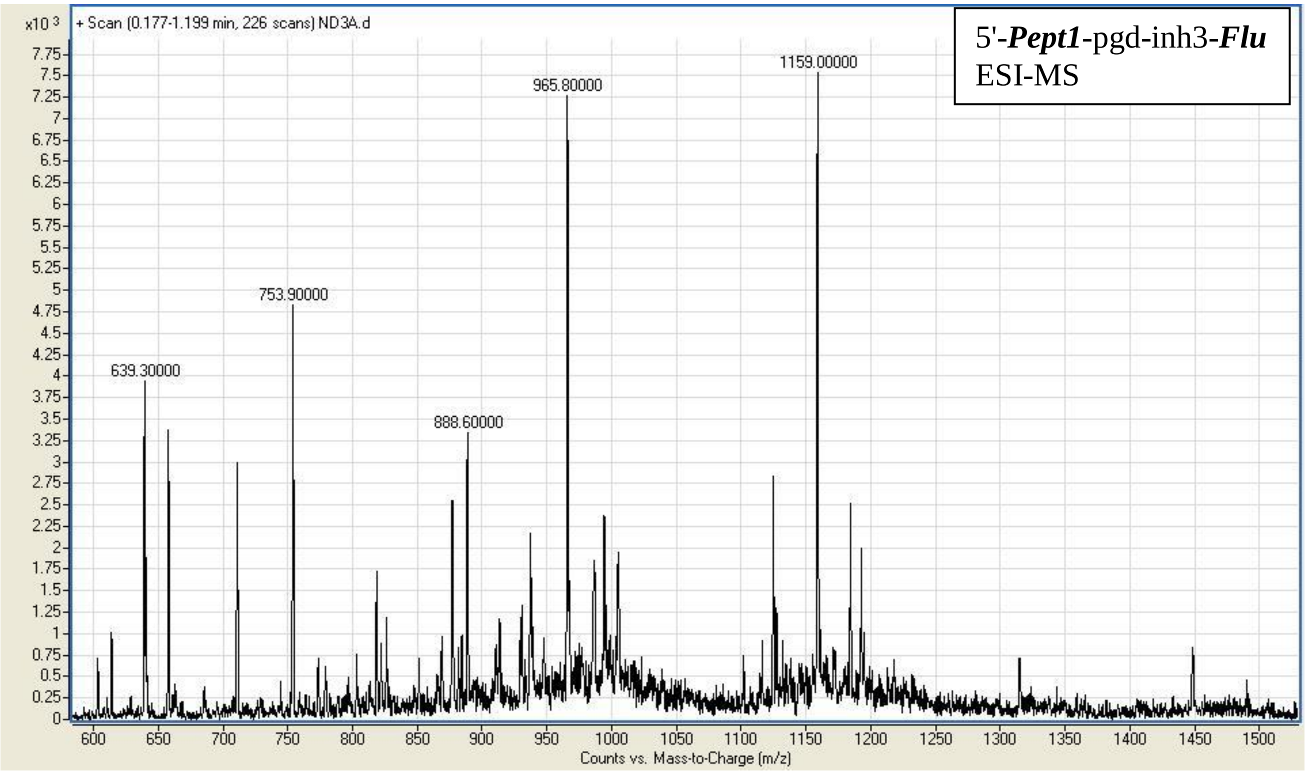
x10 3
+ Scan (0.177-1.199 min, 226 scans) ND3A.d
5'-*Pept1*-pgd-inh3-*Flu*
ESI-MS
1159.00000
965.80000
753.90000
639.30000
888.60000
Counts vs. Mass-to-Charge (m/z)

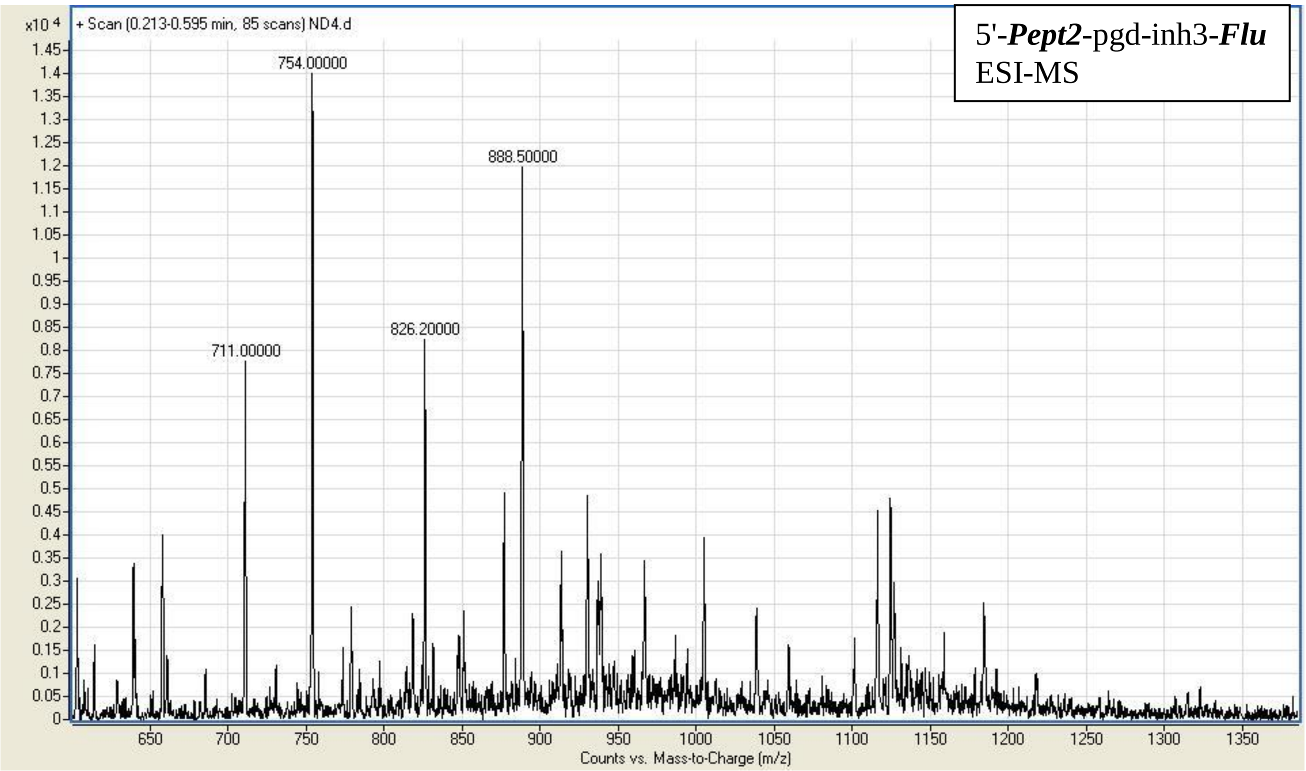
x10 4
+ Scan (0.213-0.595 min, 85 scans) ND4.d
5'-*Pept2*-pgd-inh3-*Flu*
ESI-MS
754.00000
888.50000
826.20000
711.00000
Counts vs. Mass-to-Charge (m/z)

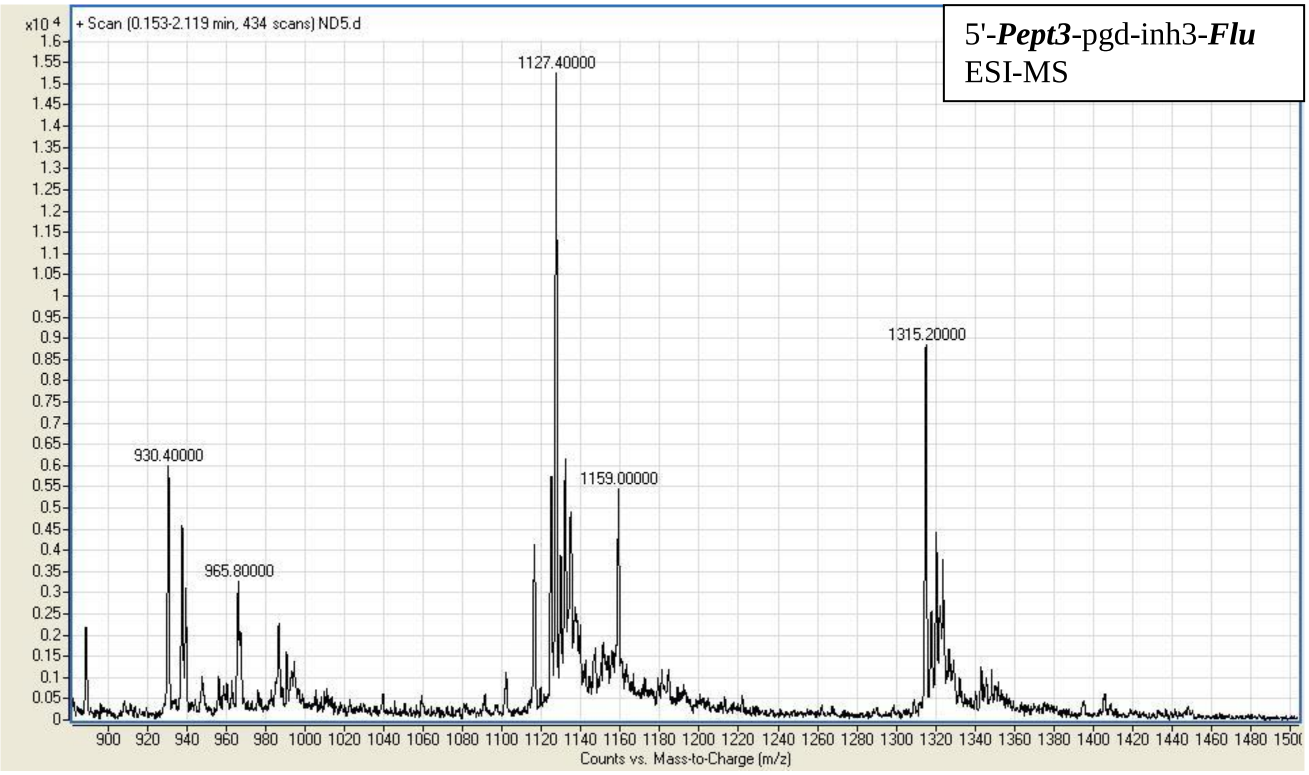
5'-***Pept3***-pgd-inh3-***Flu***
ESI-MS
+ Scan (0.153-2.119 min, 434 scans) ND5.d
x10 4
1127.40000
1315.20000
930.40000
1159.00000
965.80000
Counts vs. Mass-to-Charge (m/z)

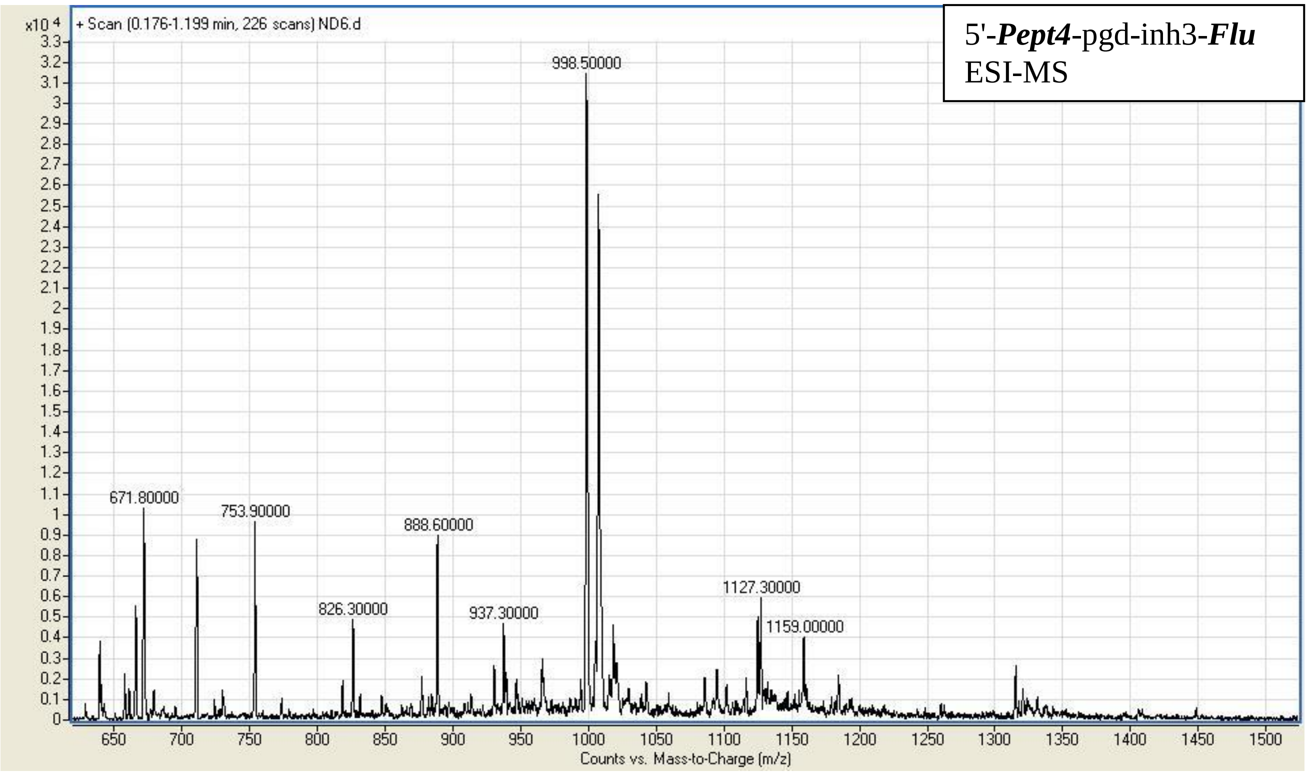
5'-***Pept4***-pgd-inh3-***Flu***
ESI-MS
+ Scan (0.176-1.199 min, 226 scans) ND6.d
x10 4
998.50000
671.80000
753.90000
888.60000
1127.30000
826.30000
937.30000
1159.00000
Counts vs. Mass-to-Charge (m/z)

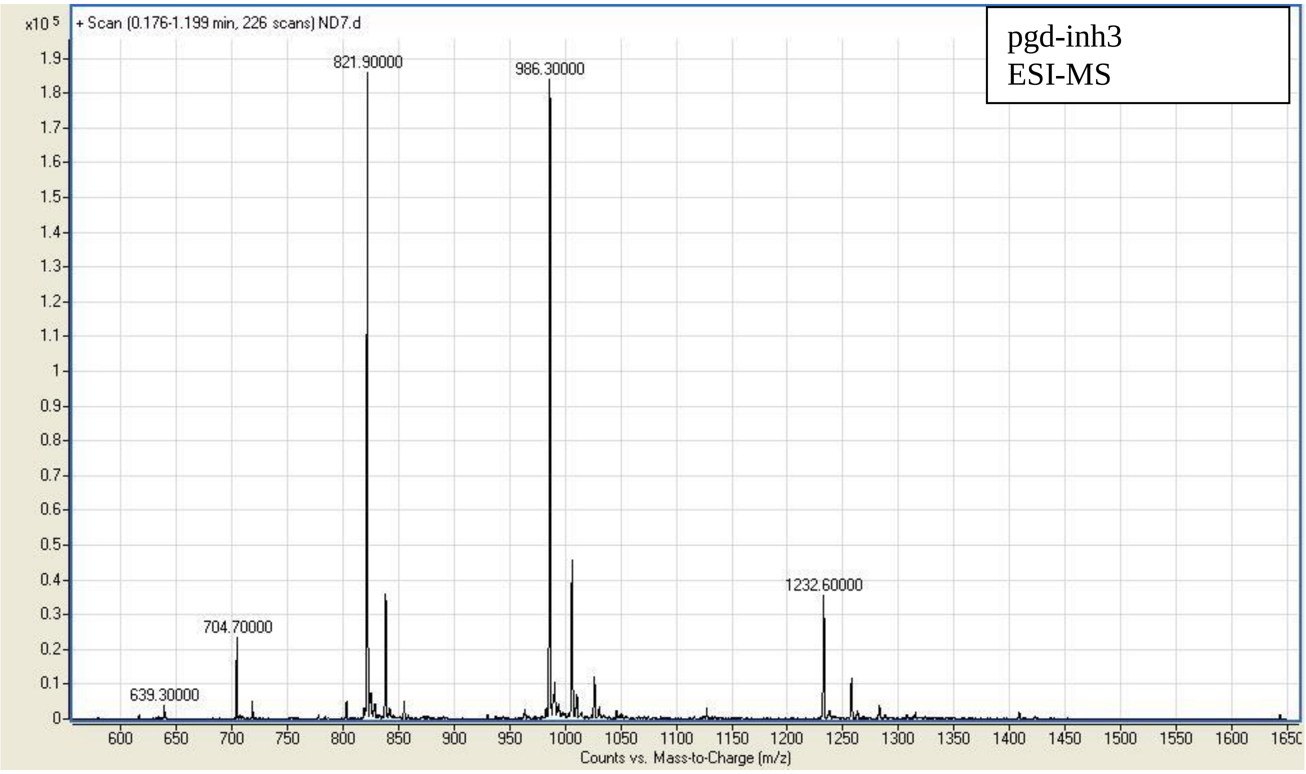
pgd-inh3
ESI-MS
x10 5
+ Scan (0.176-1.199 min, 226 scans) ND7.d
821.90000
986.30000
1232.60000
704.70000
639.30000
Counts vs. Mass-to-Charge (m/z)

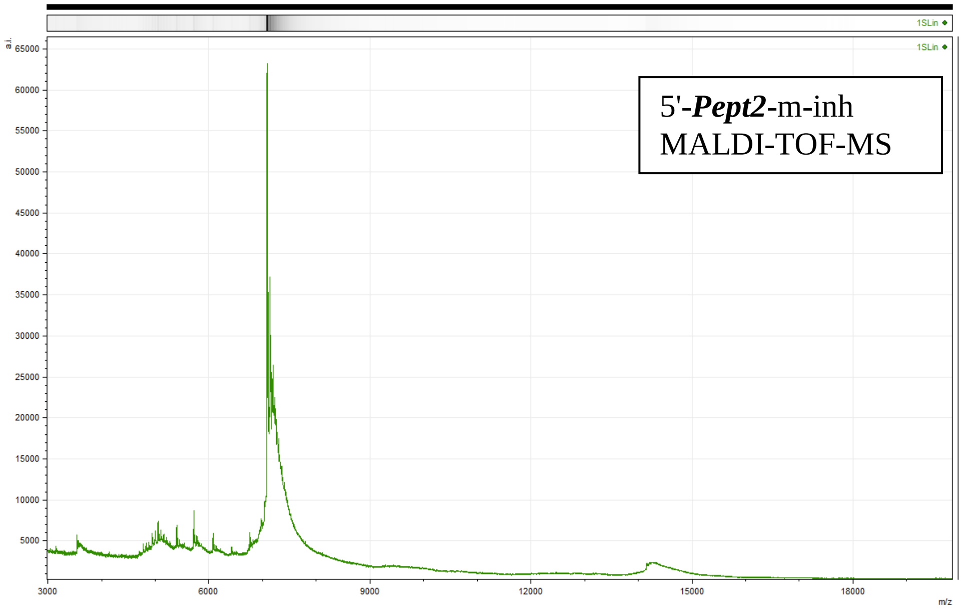
5'-Pept2-m-inh
MALDI-TOF-MS
a.i.
m/z

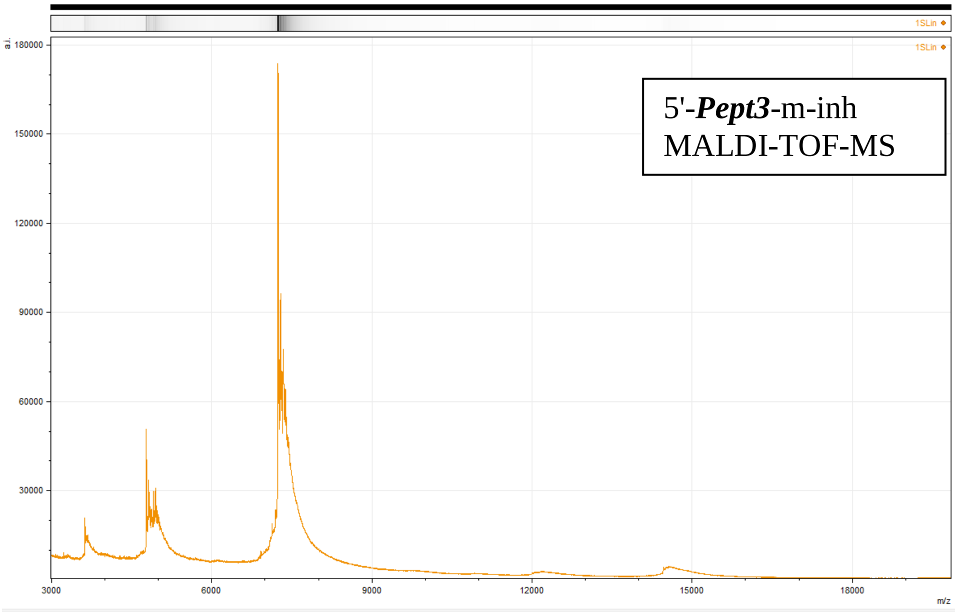
5'-*Pept3*-m-inh
MALDI-TOF-MS
a.i.
180000
150000
120000
90000
60000
30000
3000
6000
9000
12000
15000
18000
m/z
1SLin

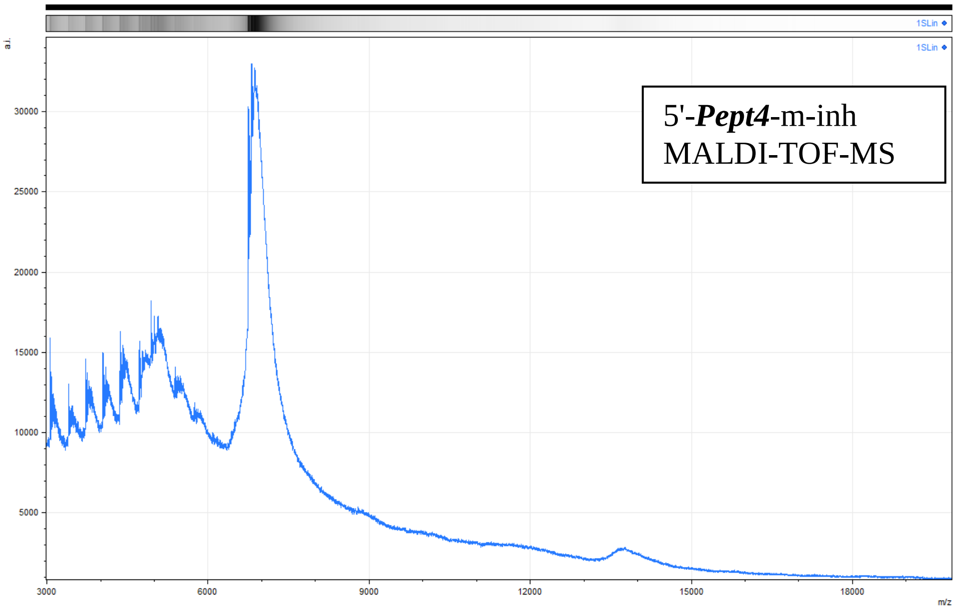
5'-*Pept4*-m-inh
MALDI-TOF-MS
a.i.
30000
25000
20000
15000
10000
5000
3000
6000
9000
12000
15000
18000
m/z
1SLin
